# Influence of working conditions on German paramedics' intention to leave the profession: a cross-sectional study

**DOI:** 10.3389/frhs.2025.1548525

**Published:** 2025-01-31

**Authors:** Thomas Hofmann, Michael Stanley, Luis Möckel

**Affiliations:** ^1^ Department of Health and Social Sciences, HSD Hochschule Döpfer GmbH, University of Applied Sciences, Potsdam, Germany; ^2^ German Society of Paramedic Sciences (DGRe), Aachen, Germany; ^3^ Faculty of Health, University Witten/Herdecke, Witten, Germany; ^4^ Offshore Rescue, Johanniter-Unfall-Hilfe e. V., Berne, Germany

**Keywords:** paramedic, working conditions, job satisfaction, commitment, intention to leave, retention

## Abstract

**Introduction:**

It is well known that Germany's ambulance service (AS) suffers from a shortage of qualified personnel, which may increase in the following years. For this reason, this study aimed to determine the percentage of AS staff considering leaving their profession and to analyse the possible causes.

**Methods:**

A self-developed questionnaire and the Employee Experience Questionnaire (EXQ) were used for this cross-sectional study.

**Results:**

A total of 814 AS staff with a mean age of 35.71 [standard deviation (SD) 9.78] years were included in this study. Immediately leaving the AS was the intention of 17.27% of participants, with 2.86% having already resigned, 14.29% taking actions such as applying for a job, and another 14.04% specifically planning to leave the AS in the foreseeable future. Those who plan to leave the AS immediately [3.82 (SD 0.79)] showed significantly lower EXQ scores than the group who plan to stay in the EMS until retirement [4.92 (SD 0.87); p_Tukey_ ≤0.001]. At the same time, it is noticeable that EMS staff often suffer from unfavourable working conditions, such as not being granted breaks, work-related calls during free time, and over time, and significant correlations with the planned length of stay in the paramedic service and exit thoughts were identified here.

**Conclusion:**

Unfavourable working conditions further aggravate the already existing shortage of qualified personnel in the ambulance service, so measures by various decision-makers are imperative.

## Introduction

The German ambulance service is suffering from a shortage of staff (1–3). On the one hand, this shortage is caused by steadily increasing call volumes (4) and, thus growing personnel requirements (2, 5). On the other hand, there are indications that many qualified personnel leave the ambulance service shortly after training (6). The exact reasons for this remain unknown, although workload seems to play a significant role (7–9).

There is no single German ambulance service. The ambulance services typically consist of small and medium-sized companies and non-governmental organisations (NGOs) that provide ambulance services to the local area. In larger cities, the cities' Fire & Rescue Services typically also staff most ambulances, with some ambulances being staffed by other companies working in accordance with contracts made with the city or county. To a small extent, the German military also supplements the ambulance service with ambulances to enable its medical staff to treat emergency patients as a routine course of work. The ambulance service is funded to the most significant part by health insurance companies, which cover the treatment provided by the ambulance service. Health insurance is generally mandatory in Germany, and the state supports people who cannot afford it. The Federal Republic of Germany consists of 16 federal states with 16 distinct ambulance service laws (Rettungsdienstgesetze). The laws which regulate current paramedic training (three years) and registration are at a national level. While the training of emergency medical technicians is not regulated by law, there is a general consensus that EMT training should be no shorter than 520 h. Before paramedic law changed in 2014, paramedic training took two years, and there are still some paramedics working in the ambulance services with the old qualification and skill set. Germany has employed prehospital doctors since 1970 (10), and traditionally, doctors would respond to all potentially life-threatening calls. The German ambulance service is changing. The frequency of using prehospital doctors is decreasing (11, 12). Since the last significant change of the paramedic law in 2021, German paramedics have been allowed to practice autonomously (13). In this article, the term paramedic includes all types of non-physician staff in the ambulance service.

The shortage of paramedics leads to unstaffed ambulances, which might directly increase the response times of the ambulance service. This situation represents a vicious circle-like development. Due to the staff shortage in the ambulance service, the workload increases for the remaining staff, possibly leading to more staff resigning. The workload also increases because of a lack of personnel in the other pillars of emergency or urgent care (i.e., lack of out-of-hours general practitioner coverage and understaffed emergency departments).

Apart from the workload, other factors may play a role in low staff retention. A survey investigating German paramedic students' intention to stay in the profession discovered that a lack of career development options and unfavourable working conditions are the main reasons for potential exit (6). Some explicit conditions that seem to impact work satisfaction in the German ambulance service have been identified. These are shift work, adherence to breaks/rest periods, overtime, and scope of employment (6, 14, 15). Reasons that seem to be comparable in other developed countries (16–19).

Before these issues can be resolved, it is necessary to investigate further the circumstances surrounding the lack of staff retention. Such research can help politicians, health service administrators, and ambulance service executives to find suitable solutions.

This paper will focus on the following research questions.

## Research questions

To analyse possible causes for the strained personnel situation in more detail, the following research questions will be answered:

(1) What is the proportion of active paramedics that are thinking about leaving the ambulance service (intention to leave), and how long are they planning to stay in the ambulance service (retention time)?
(2) What is the frequency of unfavourable working conditions in the ambulance service, and what is the relationship between unfavourable work conditions and intention to leave and length of stay?
(3) What is the relationship between the employee experience and factors such as undesirable working conditions, intentions to leave, and planned length of stay in the ambulance service?

## Method

To answer these research questions, a web-based cross-sectional survey was administered to German paramedics in clinical operative roles, excluding doctors, as these are usually either freelance or employed by hospitals and then rotas into prehospital work. This survey consisted of the validated employee Experience Questionnaire (EXQ), supplemented by questions about specific working conditions and sociodemographic data.

### Questionnaire

The EXQ was developed and validated by Fischer, Hüttermann and Werther in 2021. It describes crucial perceptions, attitudes, and behaviours of employees in organisations. The EXQ served as the basis for this cross-sectional study in a survey design (20). This questionnaire includes four subscales, which are all internationally published (21–24). The EXQ was used as a basis because it is originally in German, available without cost, and allows a holistic picture of employee experience. The EXQ is a relatively new questionnaire and has not been utilised in healthcare. However, other projects currently apply the EXQ in healthcare settings (25).

The EXQ questionnaire consists of four domains: job satisfaction (Cronbach's α = 0.80), organisational commitment (α = 0.91), individual (α = 0.85), and collective engagement (α = 0.77). The EXQ is rated using a seven-point scale, where 1 means very dissatisfied/strongly disagree, 4 neither dissatisfied nor satisfied/neutral, and 7 very satisfied/strongly agree (20).

In this context, job satisfaction is the emotional reaction to various aspects of working life. The satisfaction of personal needs at work directly impacts the quality and intensity of this emotional response (26). Satisfaction here refers to the work activity, colleagues, compensation, opportunities for advancement, and direct supervisor (24). Job satisfaction positively affects several work-related aspects, such as task-related performance (27). Organisational commitment describes how strongly a person identifies with an organisation, its concerns, goals, and values. Many studies demonstrate a positive relationship between effort, commitment, low turnover behaviours, and high loyalty to the employer (28–30). Individual engagement describes an individual's motivational state, that is, the extent to which employees invest their affective, cognitive, and behavioural resources in performing their work tasks (22). Thus, individual engagement substantially impacts individual work performance (31, 32). Collective engagement refers to the shared experience within a group of people, such as a team, department, or organisation, instead of individual engagement. It significantly indicates how effectively a social group mobilises its emotional, cognitive, and behavioural potential to address common challenges. It is considered a crucial measure of an organisation's overall performance capability (21, 33).

For the present study, the EXQ was supplemented with sociodemographic items (e.g., age, gender, marital status), items related to the working conditions of the ambulance services and two questions about intention to leave and anticipated length of stay (see Table 1). In addition, five specific working conditions defined as unfavourable [*statutory lunch break not granted*; *call-related overtime at the end of duty*; *scheduled overtime in duty roster*; phone *calls in spare time to accept additional shifts*; *statutory rest period between shifts (11 h) not observed*] were queried, which were to be rated by the study participants using a 7-point scale according to the frequency of occurrence (1, never; 2, very rarely; 3, rarely; 4, now and then; 5, frequently; 6, very frequently; 7, always). Statutory lunch break refers to not having a lunch break over the entire shift. There was no recall time given for reporting these working conditions.

**Table 1:** Sample questions from the questionnaire.

Question: How do you see your future in the ambulance service?		Question: How much longer do you intend to work in the ambulance service?	
1	I will remain in the ambulance service until further notice	1	I want to leave it as soon as possible.
2	I keep toying with the idea of leafing the ambulance service	2	Up to 5 years
3	I am planning to leave the ambulance service in the foreseeable future.	3	Up to 10 years
4	I am already taking steps (e.g., job applications, training) to leave the ambulance service.	4	Up to 20 years
5	I have already given my notice and will be leaving the ambulance service soon.	5	Until retirement

During the pre-test of the complete questionnaire in August 2022, it was checked for general comprehensibility, correctness, and face validity.

### Study participants

The questionnaire was published online via the SosciSurvey platform from 29.08.2022 to 07.10.2022 and was addressed to about 85,000 employees in German ambulance services (34). The anticipated completion time was stated as 5–10 min. Participants had to confirm their consent to participate, data collection and processing, and belonging to the target group to access the questionnaire. The target group were individuals who (i) are employed in the ambulance service for remuneration (no volunteers) or training, (ii) are working in clinical operative roles at least 80% of their time, and (iii) do not have any management roles in the ambulance service.

The online questionnaire was primarily promoted by the authors via snowball-sampling and the German Society of Paramedic Science (Deutsche Gesellschaft für Rettungswissenschaften e. V.) in social networks (Facebook, Twitter, LinkedIn, and Instagram).

The HSD Hochschule Döpfer ethics committee in Cologne raised no objections to the conduction of the research project (application reference: BEth_26_22 of 18.07.2022).

### Statistical evaluation

Percentages for categorical variables and the mean (M) and standard deviation (SD) for metric variables were calculated to represent the characteristics of the study participants. To evaluate the EXQ questionnaire, non-weighted means were first determined for the study participants' total score and individual domains (20). Based on this, mean scores were calculated for the entire sample. Since the items on the undesirable working conditions were asked using a 7-point ordinal scale, the median and interquartile range (IQR) were calculated for evaluation.

Correlations between the EXQ or the domains of the EXQ and various metric or ordinal scaled variables were analysed using Spearman correlation with the calculation of *rho* and associated 95% confidence intervals (95% CI). ANOVA was used to explore whether there were differences in the mean values of the overall EXQ and the domains when stratified by the response options for intentions to leave, length of stay, and scope of employment, respectively. If there was a significant result in the ANOVA, a post-hoc analysis was performed to calculate adjusted *p*-values (p_tukey_). Associations between intention to leave or planned length of stay in the ambulance service with unfavourable working conditions were analysed using the Kruska-Wallis test.

A *p*-value of ≤0.05 was considered statistically significant. Statistical analysis was performed using the JASP program (JASP Team, version 0.17.2).

## Results

### Characteristics of the study participants and working conditions

814 study participants gave informed consent to participate in the study and met the three (i–iii) predefined inclusion criteria. To complete the questionnaire, participants took a mean of 5:43 min (SD: 2:39).

The mean age of the study participants was 35.71 (SD 9.78) years; the majority were male (79.61%), 83.15% of participants reported 100% employment, and 50.75% worked 12-hour shift type. Most study participants reported a weekly work schedule of 48 h (30.28%) and worked an average of 13.17 (SD 17.40) hours of overtime per week in the ambulance service. A detailed description of the characteristics of the study participants is shown in Table 2.

**Table 2:** Characteristics of study participants; M, mean; SD, standard deviation.

Characteristic	*N* = 814
Age—years	M 35.71 (SD 9.78)
<20	0.25%
20–29	31.70%
30–39	33.54%
40–49	23.96%
50–59	9.46%
≥60	1.11%
Gender	Men: 79.607% (*n* = 648)
	Women: 20.147% (*n* = 164)
	Non-binary: 0.246% (*n* = 2)
Marital status	Single without child: 27.37% (*n* = 223)
	Fixed relationship without child: 35.27% (*n* = 287)
	Single parent: 2.34% (*n* = 19)
	Fixed relationship with child: 32.06% (*n* = 261)
	Other: 2.96% (*n* = 24)
Full-time equivalent	Up to 100%: 83.15% (*n* = 677)
	Up to 75%: 5.12% (*n* = 42)
	Up to 50%: 11.65% (*n* = 95)
Work experience—years	M 13.68 (SD 13.22)
Qualification	Emergency Medical Technician: 20.12% (*n* = 164)
	Paramedic (2 yrs training): 4.69% (*n* = 38)
	Paramedic (3 yrs training): 71.36% (*n* = 581)
	Paramedic Student (1st year): 0.12% (*n* = 1)
	Paramedic Student (2nd year): 0.49% (*n* = 4)
	Paramedic Student (3rd year): 0,86% (*n* = 7)
	Others: 2.35% (*n* = 19)
Shift type	No shift work: 1.63% (*n* = 13)
	8 h: 5,89% (*n* = 48)
	12 h: 50,75% (*n* = 413)
	24 h: 18,42% (*n* = 150)
	Different length: 20.05% (*n* = 163)
	Other: 3.26% (*n* = 27)
Weekly working hours of full-time employees	<38 h: 0,63% (*n* = 5)
	38h: 6,03% (*n* = 49)
	39 h: 12,31% (*n* = 100)
	40 h: 10,43% (*n* = 85)
	41 h: 1,38% (*n* = 11)
	42 h: 7.66% (*n* = 62)
	43 h: 2,14% (*n* = 17)
	44 h: 2,26% (*n* = 19)
	45 h: 21,11% (*n* = 172)
	46 h: 2,14% (*n* = 17)
	47 h: 0,38% (*n* = 3)
	48 h: 30,28% (*n* = 247)
	>48 h: 3,27% (*n* = 27)
Overtime per week	M 13.17 h (SD 17.40)

**Figure 1:**
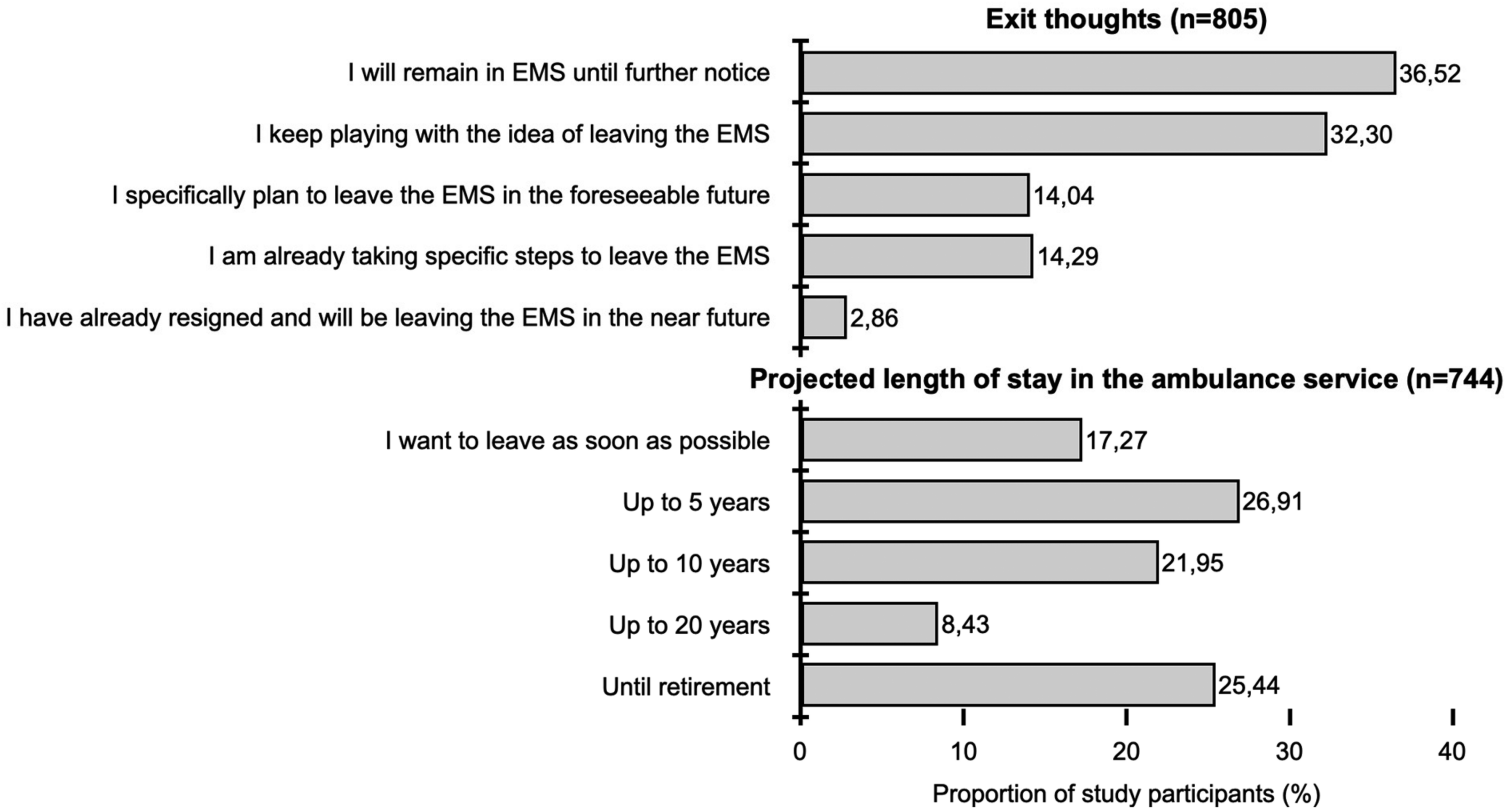
Analysis of the study participants according to intention to leave from the ambulance service and the duration they intend to work in the ambulance service.

Only 36.52% of the study participants intend to remain in the ambulance service, 14.04% plan to leave the ambulance service in the foreseeable future, another 14.29% are already taking measures (e.g., application, training) to leave the ambulance service, and 2.86% have already resigned (Figure 1). When asked how long study participants plan to remain in the ambulance service, 17.27% responded they plan to leave as soon as possible, 26.91% up to 5 years, and 21.95% up to 20 years (Figure 1). Only 25.44% of the study participants can imagine working in the ambulance service until retirement (which is typically at the age of 67 in Germany currently).

**Figure 2:**
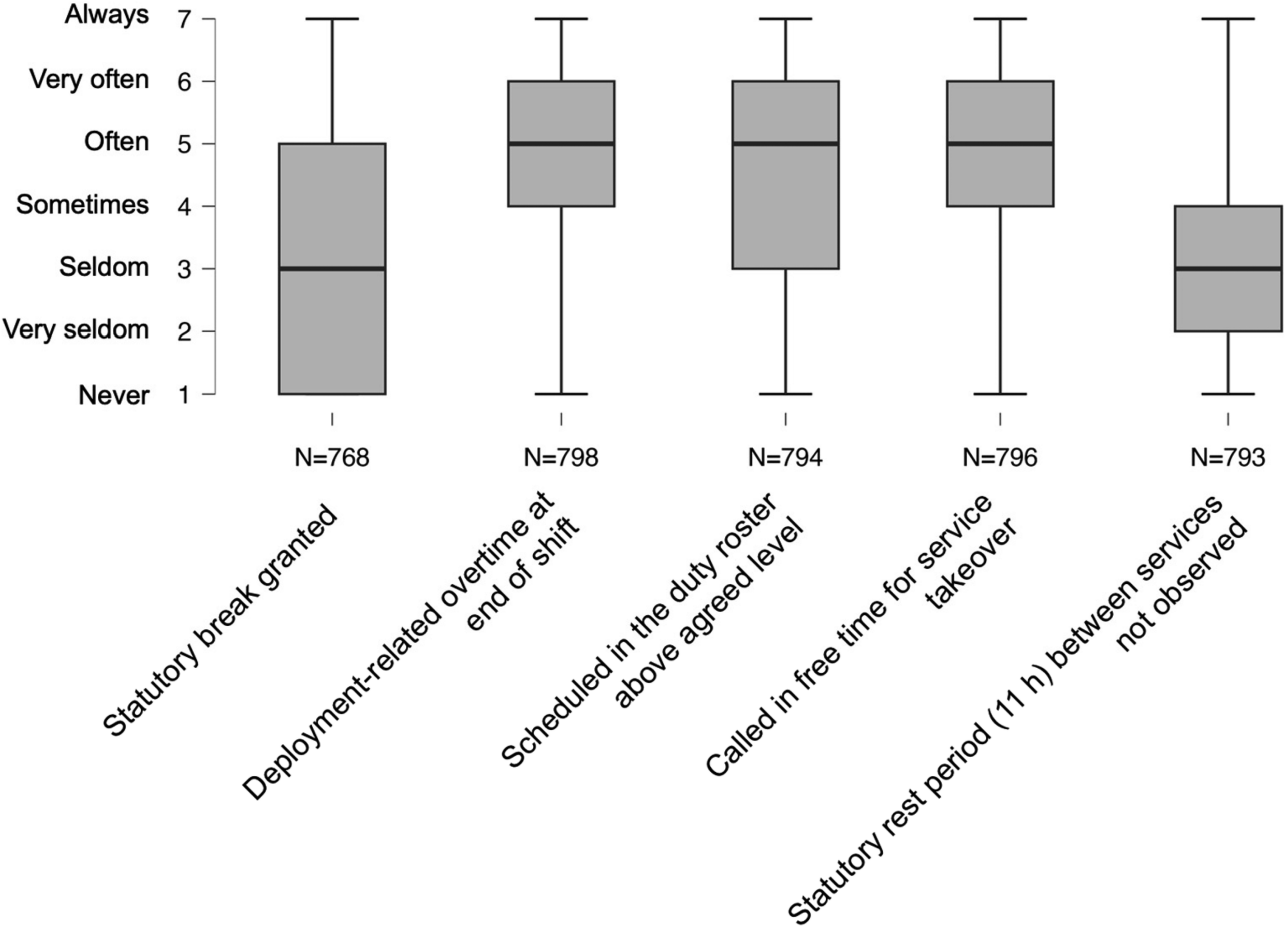
Boxplots on the frequency of unfavorable working conditions in ambulance services.

The study participants described unfavourable working conditions as follows (Figure 2). The granting of statutory lunch breaks on duty was reported by the study participants with a median value of 3 (IQR: 4.00), which is equivalent to *rare*. Overtime at the end of duty due to deployment (median: 5.00; IQR: 2.00), being scheduled to work more than agreed upon (median: 5.00; IQR: 3.00), and being called to take additional work during off hours (median: 5.00; IQR: 2.00) occurred *frequently* among study participants. The statutory rest periods of 11 h between services were not observed from *time to time* among the study participants (median: 3.00; IQR: 2.00).

Kruska-Wallis tests showed significant differences in the median values of unfavourable working conditions when examined by intention to leave (*p* ≤ 0.001) or planned length of stay in the ambulance service (≤0.003) (Table 3). A trend can be seen that those with stronger intentions to leave and shorter planned lengths of stay in the ambulance service are less frequently granted the statutory lunch break and, at the same time, more often have to work overtime at the end of duty, are more frequently over planned in the duty roster, are more frequently called for duty takeovers in their free time, and are more frequently unable to observe the statutory rest periods between duties (Table 3).

**Table 3:** Analysis of intentions to leave and length of stay in the ambulance service (AS) with the frequency of unfavourable working conditions; *p*-value determined by Kruska-Wallis test.

	Statutory lunch break granted		Overtime at the end of the shift		Scheduled in the duty roster above agreed level		Called in free time for service takeover		Statutory rest period between services (11 h) not observed	
	Median^b^ (IQR)	*p*-value	Median^b^ (IQR)	*p*-value	Median^b^ (IQR)	*p*-value	Median^b^ (IQR)	*p*-value	Median^b^ (IQR)	*p*-value
Intentions to leave^a^										
(1) Remain (*n* = 268–288)	4,00 (4,00)	*p* ≤ 0,001	4,00 (1,00)	*p* ≤ 0,001	4,00 (2,00)	*p* ≤ 0,001	4,00 (2,00)	*p* ≤ 0,001	2,00 (3,00)	*p* ≤ 0,001
(2) Thought to leave (*n* = 250–256)	3,00 (3,00)		5,00 (2,00)		5,00 (2,00)		5,00 (2,00)		3,00 (2,00)	
(3) Concrete planning (*n* = 109–112)	2,00 (2,00)		5,00 (2,00)		5,00 (2,00)		5,00 (2,00)		3,50 (3,00)	
(4) Measures to leave (*n* = 110–113).	3,00 (3,00)		5,00 (1,00)		5,00 (2,00)		6,00 (1,00)		3,50 (3,00)	
(5) Terminated (*n* = 22)	2,00 (3,50)		5,00 (1,00)		5,00 (2,75)		6,00 (1,00)		4,00 (2,00)	
Assumed length of stay in the AS										
(1) leave as soon as possible (*n* = 121–126)	2,00 (3,00)	*p* ≤ 0,001	5,00 (1,00)	*p* ≤ 0,001	5,00 (2,00)	*p* = 0,003	6,00 (1,00)	*p* ≤ 0,001	4,00 (3,00)	*p* ≤ 0,001
(2) Up to 5 years (*n* = 194–199)	2,00 (3,00)		5,00 (2,00)		5,00 (2,00)		5,00 (2,00)		3,00 (2,00)	
(3) Up to 10 years (*n* = 156–162)	3,00 (4,00)		5,00 (1,00)		5,00 (3,00)		5,00 (2,00)		3,00 (2,00)	
(4) Up to 20 years (*n* = 57–61)	4,00 (4,00)		5,00 (1,00)		4,00 (2,00)		4,00 (3,00)		2,00 (3,00)	
(5) Until retirement (*n* = 176–187).	4,00 (5,00)		4,00 (1,00)		4,00 (3,00)		4,00 (2,00)		2,00 (3,00)	

^a^ (1) I will remain in the ambulance service for the time being; (2) I keep toying with the idea of leaving the ambulance service; (3) I have concrete plans to leave the ambulance service in the foreseeable future; (4) I am already taking steps (e.g., applications, training) to leave the ambulance service. (5) I have already resigned and will leave the ambulance service in the near future.

^b^ 1, never; 2, very seldom; 3, seldom; 4, now and then; 5, frequently; 6, very frequently; 7, always.

### Satisfaction, commitment, and engagement of participating paramedics

The overall EXQ was M 4.52 (SD 0.88) for participants, as well as job satisfaction M 4.32 (SD = 1.03), organisational commitment M 4.12 (SD 1.60), individual engagement M 5.53 (SD 0.92), and collective engagement M 3.95 (SD 1.14).

The total EXQ score and the domains of job satisfaction and commitment showed significant inverse correlations (*p* ≤ 0.001) with overtime per week, *assignment-related overtime at the end of shifts*, *scheduling in the duty roster beyond the agreed level*, *contacting in free time to take over shifts,* and *not taking statutory rest between duties* (*rho*: −0.18 to −0.29) (Table 4). Furthermore, significant inverse correlations were present between collective commitment and overtime hours per week, assignment-related overtime hours at the end of duty, scheduling in the duty roster beyond what was agreed upon, and contacting during off-duty hours for duty takeovers (*rho*: −0.10 to −0.18; *p* ≤ 0.004). Since these are negative correlations, lower values of the EXQ and the domains occur the more overtime is worked and the more frequently the undesirable work conditions arise. The work condition statutory *lunch break granted was* correlated with the total EXQ score as well as all four domains (*rho*: 0.16 to 0.29; *p* ≤ 0.001), which means that the less often statutory lunch breaks were granted, the lower the total EXQ as well as the four domains of the EXQ.

**Table 4:** Correlations between sociodemographic/occupational factors and unfavourable working conditions with the overall EXQ and individual domains (*n* = 761–813); marked in bold are all correlations with *rho* ≥*0*.1 and *p* ≤ 0.05.

Correlated variables	Total EXQ		Job satisfaction		Organisational commitment		Individual engagement		Collective engagement	
	*Rho*	*p*-value	*Rho*	*p*-value	*Rho*	*p*-value	*Rho*	*p*-value	*Rho*	*p*-value
Age—years	0.01	0.853	0.02	0.579	0.09	0.013	−0.07	0.057	−0.01	0.823
Weekly working time	−0.09	0.008	−0.07	0.037	−0.03	0.489	−0.09	0.010	−0.08	0.018
Overtime/week	**−0**.**19**	**≤0**.**001**	**−0**.**18**	**≤0**.**001**	**−0**.**18**	**≤0**.**001**	−0.02	0.620	**−0**.**18**	**≤0**.**001**
Work experience—years	−0.07	0.065	−0.08	0.030	0.04	0.312	−0.09	0.008	−0.06	0.094
Statutory lunch break granted	**0**.**28**	**≤0**.**001**	**0**.**29**	**≤0**.**001**	**0**.**24**	**≤0**.**001**	**0**.**17**	**≤0**.**001**	**0**.**16**	**≤0**.**001**
Overtime at the end of the shift	**−0**.**19**	**≤0**.**001**	**−0**.**26**	**≤0**.**001**	**−0**.**22**	**≤0**.**001**	−0.02	0.674	**−0**.**10**	**0**.**004**
Scheduled in the duty roster above agreed level	**−0**.**21**	**≤0**.**001**	**−0**.**23**	**≤0**.**001**	**−0**.**24**	**≤0**.**001**	−0.08	0.028	**−0**.**12**	**≤0**.**001**
Called in free time for service takeover	**−0**.**22**	**≤0**.**001**	**−0**.**29**	**≤0**.**001**	**−0**.**24**	**≤0**.**001**	−0.03	0.345	**−0**.**12**	**≤0**.**001**
Statutory rest period between services (11 h) not observed	**−0**.**18**	**≤0**.**001**	**−0**.**23**	**≤0**.**001**	**−0**.**22**	**≤0**.**001**	−0.07	0.041	−0.05	0.203

The ANOVA (Table 4) showed significant differences in the mean values of the total EXQ (*p* = 0.049), in the domains commitment (*p* = 0.034) and individual engagement (*p* = 0.009) if these were stratified according to the scope of employment, whereby the group with up to full-time equivalent always showed higher values than the group with up to 75% time equivalent. When stratifying by intention to leave and the length of stay undertaken, ANOVA for the overall EXQ and all four domains showed significant differences in the mean values of the respective subgroups. In the case of intention to leave, it can be observed that the higher the urge to leave the ambulance service, the lower the scores. Comparable is the length of stay, the longer the participants think to stay in the ambulance service, the higher the score of the total EXQ and the four domains of the EXQ. The detailed list of post-hoc analyses is shown in Table 5.

**Table 5:** ANOVAs and post-high analyses (p_Tukey_) of the overall EXQ and the domains by full-time equivalent, intentions to leave, and length of stay made in the ambulance service (AS).

	Total EXQ		Job satisfaction		Organisational commitment		Individual engagement		Collective engagement	
	ANOVA M (SD)	Post-Hoc	ANOVA M (SD)	Post-Hoc	ANOVA M (SD)	Post-Hoc	ANOVA M (SD)	Post-Hoc	ANOVA M (SD)	Post-Hoc
Full-time equivalent (1) Up to 100% (*n* = 670) (2) Up to 75% (*n* = 94) (3) Up to 50% (*n* = 42)	*p* = 0.049 4.55 (0.89) 4.20 (0.89) 4.49 (0.79)	(1) vs. (2): *p* = 0.04	*p* = 0.311 4.35 (1.05) 4.14 (0.99) 4.23 (0.99)	-	*p* = 0.034 4.17 (1.60) 3.54 (1.56) 4.01 (1.55)	(1) vs. (2): *p* = 0.034	*p* = 0.009 5.56 (0.92) 5.10 (1.06) 5.51 (0.92)	(1) vs. (2): *p* = 0.006 (2) vs. (3): *p* = 0.047	*p* = 0.568 3.95 (1.17) 3.80 (1.11) 4.02 (1.01)	-
Intentions to leave^a^ (1) Remain (*n* = 294) (2) Thought to leave (*n* = 260) (3) Concrete planning (*n* = 113) (4) Measures to leave (*n* = 114) (5) Terminated (*n* = 23)	*p* ≤ 0.001 5.00 (0.80) 4.39 (0.74) 4.29 (0.84) 3.90 (0.83) 4.13 (0.83)	(1) vs. all: *p* ≤ 0.001 (2) vs. (4): *p* ≤ 0.001 (3) vs. (4) *p* = 0.002	*p* ≤ 0.001 4.91 (0.93) 4.15 (0.91) 3.88 (0.93) 3.75 (0.97) 3.78 (0.95)	(1) vs. all: *p* ≤ 0.001 (2) vs. (4): *p* ≤ 0.001	*p* ≤ 0.001 4.79 (1.57) 3.92 (1.34) 3.98 (1.60) 3.13 (1.53) 3.17 (1.46)	(1) vs. all: *p* ≤ 0.001 (2) vs. (4): *p* ≤ 0.001 (3) vs. (4): *p* ≤ 0.001	*p* ≤ 0.001 5.90 (0.75) 5.45 (0.88) 5.32 (1.05) 4.97 (0.95) 5.52 (0.76)	(1) vs. (2), (3), (4): *p* ≤ 0.001 (2) vs. (4) *p* ≤ 0.001 (3) vs. (4): *p* = 0.02 (4) vs. (5) *p* = 0.045	*p* ≤ 0.001 4.31 (1.10) 3.84 (1.11) 3.80 (1.11) 3.47 (1.12) 3.65 (1.20)	(1) vs. all: *p* ≤ 0.049 (2) vs. (4): *p* = 0.027
Assumed length of stay in the AS (1) Leave as soon as possible (*n* = 129) (2) Up to 5 years (*n* = 200) (3) Up to 10 years (*n* = 164) (4) Up to 20 years (*n* = 63) (5) Until retirement (*n* = 190)	*p* ≤ 0.001 3.82 (0.79) 4.35 (0.79) 4.61 (0.69) 5.05 (0.81) 4.92 (0.87)	(1) vs. all: *p* ≤ 0.001 (2) vs. all: *p* ≤ 0.016 (3) vs. all: *p* ≤ 0.003	*p* ≤ 0.001 3.50 (0.84) 4.11 (0.92) 4,42 (0.89) 4,94 (0.98) 4,79 (1.01)	(1) vs. all: *p* ≤ 0.001 (2) vs. all: *p* ≤ 0.014 (3) vs. all: *p* ≤ 0.002	*p* ≤ 0.001 3.11 (1.50) 3.87 (1.44) 4.22 (1.45) 4.67 (1.58) 4.78 (1.62)	(1) vs. all: *p* ≤ 0.001 (2) vs. (4), (5): *p* ≤ 0.003 (3) vs. (5): *p* ≤ 0.005	*p* ≤ 0.001 5.00 (1.02) 5.39 (0.94) 5.62 (0.71) 6.06 (0.74) 5.81 (0.87)	(1) vs. all: *p* ≤ 0.001 (2) vs. (4), (5): *p* ≤ 0.001 (3) vs. (4): *p* = 0.008	*p* ≤ 0.001 3.39 (1.15) 3.83 (1.09) 4.02 (1.04) 4.38 (1.10) 4.24 (1.18)	(1) vs. all: *p* ≤ 0.004 (2) vs. (4), (5): *p* ≤ 0.007

^a^ (1) I will remain in the ambulance service until further notice; (2) I keep toying with the idea of leaving the ambulance service; (3) I have concrete plans to leave the ambulance service in the foreseeable future; (4) I am already taking steps (e.g., applications, training) to leave the ambulance service; (5) I have already given notice and will leave the ambulance service in the near future.

## Discussion

This study aimed to answer the research questions about paramedics' intention to leave, retention time, and the correlation between unfavourable working conditions and their interrelationships.

Based on the results, it could be said that work conditions require improvement. Unfavourable conditions, such as lack of breaks, unpredictable ends of duty, work-related phone calls during free time, and utilisation beyond the contractually agreed work hours, seem widespread among those who answered the questionnaire. These circumstances are significantly related to the presumed length of stay on the job and the concrete intention to leave. It can be described as alarming that 63.58% of the respondents are at least latently considering a career change. In the survey, 31.19% of the respondents actively sought an exit strategy from the ambulance service. 2.86% had already resigned. Almost 2/3 consider leaving the ambulance service, which means a significant increase to 2018 when 54% were thinking about it (7).

Compared to the study of Hofmann and Macke (6), which asked about the planned length of stay in the ambulance service among paramedic students, the proportion of those who want to stay in service until retirement (27.44% vs. 25.44%) is roughly the same. Of note, in the paper, as mentioned earlier, only 2.68% of paramedics in training want to leave the ambulance service immediately after completing paramedic training. In the present study, on the other hand, with staff who have already been working in the ambulance service for an average of 13.68 years, 17.27% want to leave as soon as possible (Figure 1).

It is noticeable that the different domains of the EXQ have very different characteristics. In particular, individual engagement is always the domain with the highest scores in all approaches, both in the overall sample and when stratified by intentions to leave and length of stay (Table 5) and is mainly independent of unfavourable work circumstances (see Table 4). Although the mean values of the score for individual engagement differed significantly in the subgroups for expected length of stay and intentions to leave, the differences in the mean values are relatively small (Table 5). This could be interpreted as an indication that employees in the ambulance service have a high level of individual engagement despite adverse circumstances and thus ensure good patient care with a great deal of passion.

In addition to the effects on the length of stay and intentions to leave, poor working conditions also impact the health of paramedics. In particular, the high working time burden is associated with an increased prevalence of depression (35). High levels of work time led to more frequent sick leave (36) and increased the occurrence of occupational accidents (37).

With specific working conditions, this study adds a relevant aspect to the ongoing international discussion about paramedics' retention, mainly focusing on ambulance calls or health-related aspects (9, 38).

### Limitations

This study has several limitations. First, as a cross-sectional study, the results are vulnerable to selection and recall bias. However, since 294 study participants indicated that they intended to remain in the ambulance service until further notice, a sufficient proportion of the satisfied workforce should have participated to allow meaningful conclusions to be drawn. Additionally, it is possible that the respondents answered questions about working conditions spontaneously, without consideration given to reflecting emotional responses. For example, in the case of the question about weekly overtime, it cannot be assumed that this is an adequate assessment but rather a perceived truth. Furthermore, as the survey asked about what impacts employee experiences negatively, participants focused on the negative job aspects. This might have led to a negative influence on their responses—especially paramedics who strongly consider leaving ambulance service may have contributed to the recall bias.

As this study has a cross-sectional design, no causal conclusions are possible.

Given the small-cell and regionally very different organisation types of the German ambulance service there might be confounders impacting the results we had not identified; for instance, kind of employer (i.e., military, private, non-governmental organisation, regional differences).

Finally, it is essential to note that the survey asked for the intention to leave and the anticipated length of stay. It is impossible to conclude whether the intention to leave is being realised nor if the expected length of stay is adhered to.

## Conclusions

The personnel situation is tense, and the survey results indicate that the problem could worsen. Almost 2/3 of the respondents are latently considering leaving the ambulance service. A strong correlation exists between selected unfavourable work conditions and intention to leave the ambulance service. There is a need for timely, concerted action by employers, unions, and politicians to counter these dramatic prospects for the future decisively.

## Data Availability

The datasets presented in this article are not readily available due to German and European data regulations. Requests to access the datasets should be directed to t.hofmann@hs-doepfer.de.
